# ADAPTING THE ECHO MODEL TO MEET THE NEEDS OF RURAL DEMENTIA CAREGIVERS

**DOI:** 10.1093/geroni/igac059.887

**Published:** 2022-12-20

**Authors:** Sabine Schenck, Catherine Carrico, Stacy Carling

**Affiliations:** University of Wyoming, Laramie, Wyoming, United States; University of Wyoming, Laramie, Wyoming, United States; University of Wyoming, Laramie, Wyoming, United States

## Abstract

Caring for an individual with dementia may result in caregiver stress and burden (Cassie & Sanders, 2008), which can lead to detrimental health outcomes if not managed with effective coping skills and support (Shulz & Martire, 2004). Access to support and psychoeducation is limited in rural and frontier communities, and solutions are needed to reach caregivers across sparsely populated regions. Project ECHO (Extension for Community Health Outcomes) is an evidence-based model designed to improve patient outcomes through healthcare provider education. The Wyoming Dementia Together Caregiver Network is the first of its kind to adapt the ECHO model for family caregivers of persons with dementia. This presentation details evaluation results of 24 session across 52 weeks (n=162). Results suggest that adaptation of Project ECHO for family caregivers is feasible and palatable. In addition, the adapted program shows promise for improving caregiver outcomes such as depressive symptoms and caregiver burden.

